# Dynamic progression of Wernicke encephalopathy in a gastric cancer patient: Multimodal MRI insights with arterial spin labeling perfusion imaging

**DOI:** 10.1097/MD.0000000000043580

**Published:** 2025-08-08

**Authors:** Zhigang Li, Qicheng Min, Jiadi Chen, Boyong Chen, Wenming Chen, Juxiang Su, Lihui Zhu

**Affiliations:** a Neurology Center, Guangzhou Fosun Chancheng Hospital of Guangdong Pharmaceutical University, Guangzhou, China; b Department of Neurology, Tongji Hospital, Tongji Medical College, Huazhong University of Science and Technology, Wuhan, China.

**Keywords:** multimodal magnetic resonance imaging, Wernicke encephalopathy

## Abstract

**Rationale:**

Wernicke encephalopathy (WE), a neurological emergency caused by thiamin deficiency, is traditionally diagnosed based on the triad of ophthalmoplegia, ataxia, and confusion. However, this classic presentation occurs in fewer than 10% of cases, complicating early recognition. Untreated cases risk irreversible brain damage or progression to Korsakoff syndrome. While magnetic resonance imaging (MRI) aids diagnosis, early-stage structural abnormalities may be subtle or absent. Arterial spin labeling (ASL), a noninvasive perfusion imaging technique, offers potential for detecting microcirculatory changes preceding cytotoxic edema. This case explores ASL’s diagnostic utility in WE through a high-risk patient with atypical progression.

**Patient concerns:**

A 50-year-old female with gastric adenocarcinoma developed persistent vomiting following chemotherapy. By hospital day 9, she rapidly deteriorated into a comatose state (Glasgow Coma Scale: E1V1M3), prompting neurological evaluation. No classic WE triad features were initially documented.

**Diagnoses:**

Brain MRI revealed bilateral thalamic, periventricular, and periaqueductal gray matter hyperintensities on T2-FLAIR/DWI. ASL perfusion imaging demonstrated elevated cerebral blood flow (CBF) in these regions, extending to frontal and parietal lobes. Follow-up diffusion-weighted imaging (DWI) showed lesion progression involving cortical and medullary areas, with ASL hyperperfusion exceeding diffusion-restricted zones. Serum thiamin levels (<1 ng/mL) confirmed deficiency, establishing WE diagnosis.

**Interventions:**

Initial supportive care: Vasopressors, fluid resuscitation, methylprednisolone (40 mg/day), granulocyte colony-stimulating factor critical intervention delay: Thiamine replacement deferred until day 11 due to pending laboratory confirmation family-directed care: Against medical advice, transferred to local hospital prior to initiating high-dose IV thiamin.

**Outcomes:**

Radiological progression: Lesion expansion from deep gray matter to cortical/medullary regions within 48 hours Therapeutic uncertainty: Final neurological recovery status unreported due to care discontinuity.

**Lessons:**

ASL may identify perfusion alterations prior to DWI-detectable cytotoxic edema, suggesting a role in WE’s early diagnostic algorithm. Rapid lesion progression in high-risk patients necessitates urgent thiamin repletion even without classic symptoms. Neuroimaging findings in WE may exhibit dynamic spatial-temporal evolution, requiring multimodal imaging interpretation. Serum thiamin levels remain critical for diagnostic confirmation in radiologically ambiguous cases.

## 1. Backgrounds

Wernicke encephalopathy (WE), initially documented by Carl Wernicke in 1881, is an acute metabolic encephalopathy of the central nervous system resulting from thiamin (Vitamin B1) deficiency.^[1]^ While commonly linked to chronic alcoholism, WE can manifest in various clinical scenarios such as malignancy, gastrointestinal surgery, and persistent vomiting. Its incidence is deemed infrequent, with documented morbidity rates ranging from 0.4% to 2.8%.^[2]^

Clinically, WE is traditionally characterized by the triad of ophthalmoplegia, ataxia, and confusion. However, this complete triad is only evident in <10% of cases,^[3]^ with the majority presenting with vague symptoms like apathy, disorientation, or lethargy.^[4,5]^ These nonspecific manifestations often overlap with those of other medical conditions, particularly in oncology or intensive care contexts, leading to frequent underdiagnosis. Laboratory findings lack specificity, and serum thiamin levels may not accurately reflect the condition in its early phases. Hence, imaging plays a pivotal role in the diagnostic process.

Magnetic resonance imaging (MRI) plays a pivotal role in the diagnostic assessment of WE. Characteristic MRI manifestations encompass symmetrical T2-weighted and FLAIR hyperintensities observed in the thalami, periaqueductal gray matter, hypothalamus, mammillary bodies, and the floor of the 4th ventricle.^[4]^ Additionally, lesions may extend to the medulla, pons, red nucleus, basal ganglia, and corpus callosum. While diffusion-weighted imaging (DWI) commonly reveals hyperintensities in affected areas like the mammillary bodies, thalami, and midbrain,^[6–8]^ the apparent diffusion coefficient (ADC) values exhibit variability, ranging from elevated to decreased or within normal limits.^[9]^ This variability may mirror the dynamic interplay between cytotoxic and vasogenic edema in WE, emphasizing the necessity for supplementary imaging biomarkers.

Arterial spin labeling (ASL) MRI is a noninvasive perfusion technique used to quantify cerebral blood flow (CBF) without the need for contrast agents. This method has demonstrated potential in identifying perfusion abnormalities before structural changes occur. Despite this, there is a paucity of studies investigating ASL in WE. Bhan et al observed bilateral thalamic hyperperfusion on CT perfusion while CT angiography showed normal results.^[10]^ In a separate study, Ann et al noted increased ASL perfusion in the medial thalami, mammillothalamic tracts, and periaqueductal regions in WE, alongside cortical hypoperfusion.^[11]^ These findings suggest that perfusion imaging may detect early microvascular dysfunction preceding diffusion restriction.

Experimental studies in pyrithiamine-induced thiamin deficiency models have provided evidence for this hypothesis. Watanabe et al documented perivascular necrosis, astrocytic swelling, and petechial hemorrhage through electron microscopy, indicative of microcirculatory damage.^[12]^ These microvascular changes likely underlie the observed hyperperfusion on ASL, potentially indicating impaired autoregulation and metabolic strain.

In this case report, we describe a challenging diagnostic case of WE in a patient with gastric adenocarcinoma. The patient experienced persistent vomiting after chemotherapy and subsequently underwent transarterial embolization. We underscore the evolving nature of brain lesions and demonstrate through ASL the presence of perfusion abnormalities in areas not yet evident on conventional imaging sequences. This case offers new perspectives on the progressive imaging manifestations of WE and advocates for the potential efficacy of ASL in promptly identifying microcirculatory impairments.

## 2. Case report

A 50-year-old woman was diagnosed with mucinous adenocarcinoma of the gastric antrum and received chemotherapy. She was admitted to the oncology department after experiencing vomiting for 15 days. Upon admission, she was conscious but had frequent vomiting, poor appetite, abdominal distention, normal abdominal tension, no rebound tenderness, palpable fluid wave, and reduced bowel sounds. Due to her cancer diagnosis, she underwent superselective angiography involving perfusion of the left gastric artery and gastroduodenal artery, followed by embolization of the gastroduodenal artery.

By the 7th day, she displayed symptoms including lethargy, flat affect, poor sleep, persistent fatigue, nausea, and vomiting. By the 9th day, her level of consciousness deteriorated to stupor, necessitating transfer to the neurology department. Neurological evaluation indicated stupor with unresponsiveness to verbal stimuli (Glasgow Coma Scale: E2V2M4), sluggish pupillary light reflexes, decreased muscle tone in all limbs, and absence of neck rigidity. Assessments of motor, sensory, and cerebellar functions were hindered by lack of cooperation, rendering neurological manifestations nonspecific.

Laboratory results revealed reduced levels of white blood cells, hemoglobin, and platelets in the serum. Analysis of cerebrospinal fluid (CSF) indicated transparent fluid with normal opening pressure and white cell count. Abnormalities in the CSF included a slight elevation in total protein (517 mg/L; reference range: 100–450 mg/L), a minor increase in glucose levels (5.13 mmol/L; reference range: 2.50–4.50 mmol/L), and a marginal decrease in chloride levels (119.6 mmol/L; reference range: 120.0–132.0 mmol/L).

MR image acquisition was performed using a 1.5 T MR scanner (GE Signa HDi; GE Medical Systems, Milwaukee). ASL images were performed using a 3D pseudocontinuous ASL (pCASL) with a fast spin echo stack-of-spiral readout with 8 interleaves and the following parameters: TR/TE 4610/10.5 ms, FOV:240 mm^2^, matrix size: 64 mm^2^, and slice thickness of 4.0 mm across 34 axial slices. The acquisition employed 8 spiral arms with 512 sampling points per arm and 3 signal averages (NEX = 3). The bandwidth was set at 62.5 kHz. Pseudocontinuous spin labeling was performed for 1.525 seconds before a post–spin-labeling delay of 2 seconds. This setting resulted in acquisition of 3D voxel size of 3.8 × 3.8 × 4 mm^3^ during 4:30 minutes. Image reconstruction was performed online by the scanner software. Pairwise subtraction between label and control images was obtained and averaged to generate the mean difference and converted to CBF maps.

**Figure 1.**
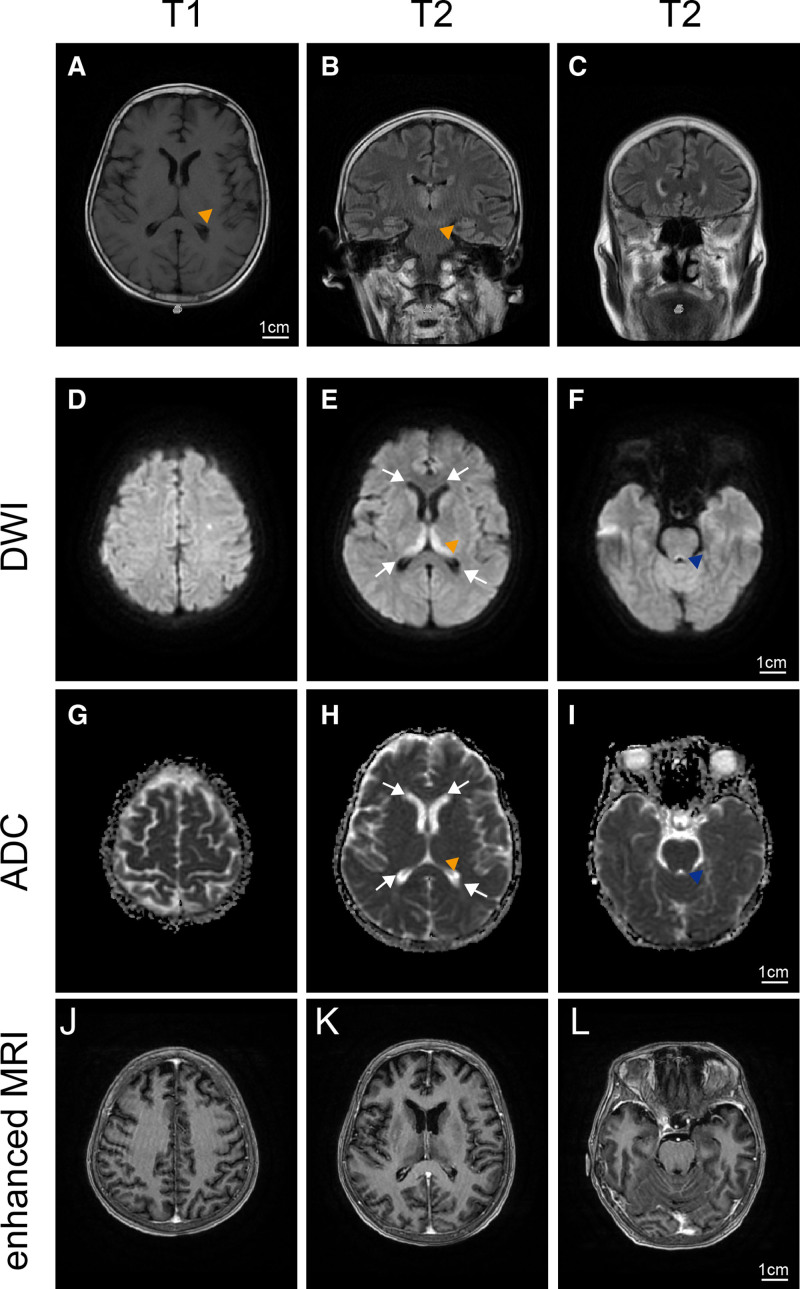
(A to L) MRI images acquired on the 9th day post admission. (A) T1 showed slightly low signal intensity in the internal bilateral thalamus. (B and C) T2 FLAIR showed high signal intensity in the internal bilateral thalamus (yellow triangle), bilateral regions adjacent to the lateral ventricles. (D) Diffusion-weighted imaging (DWI) did not demonstrate abnormal signals in bilateral frontal and parietal lobe. (E and F) DWI revealed high signal intensity in the internal bilateral thalamus (yellow triangle), bilateral caudate nucleus head (white arrows), and the periaqueductal area (blue triangle) at the posterior margin of the brainstem. (G) Apparent diffusion coefficient (ADC) did not demonstrate abnormal signals in bilateral frontal and parietal lobe. (H and I) ADC revealed slightly low signal intensity in the internal bilateral thalamus (yellow triangle), bilateral caudate nucleus head (white arrows), and the periaqueductal area (blue triangle) at the posterior margin of the brainstem. (J, K, and L) Enhanced MRI in the same regions did not show abnormal enhancement. Scale bar = 1 cm. MRI = magnetic resonance imaging.

**Figure 2.**
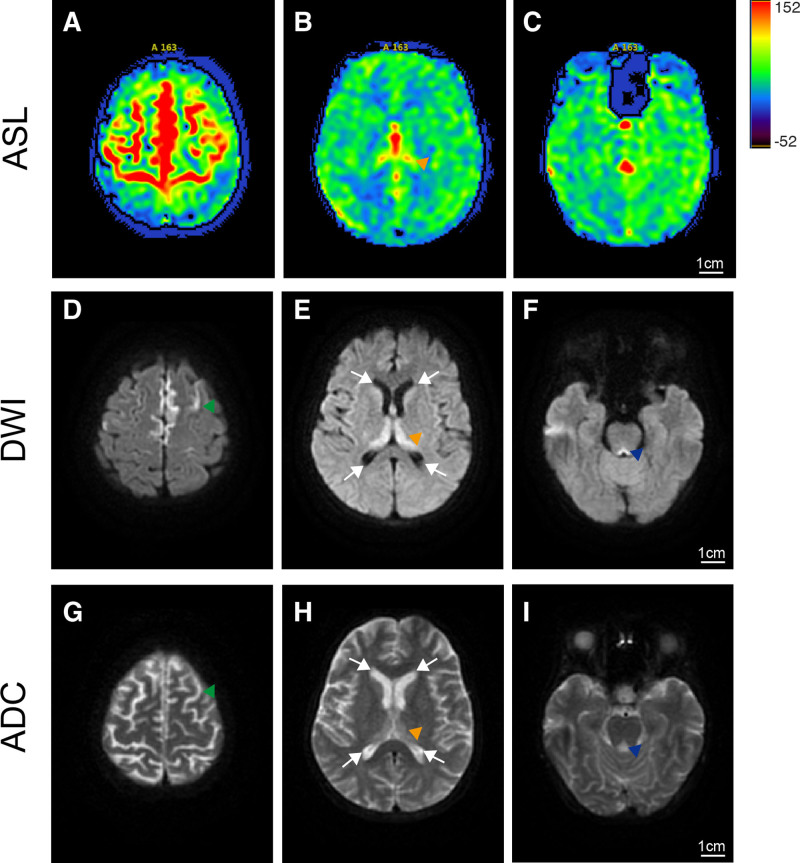
(A to I) MRI images acquired on the 10th day post admission. (A) Arterial spin labeling (ASL) revealed cerebral blood flow (CBF) increased in large areas of the bilateral frontal and parietal lobes beside the midline. Concurrently, peripheral cortical perfusion was insufficient. (B and C) ASL showed high CBF in the internal bilateral thalamus (yellow triangle) and the periaqueductal area (blue triangle) at the posterior margin of the brainstem. (D, E, and F) DWI demonstrated high signal intensity in surrounding areas of the midline of the bilateral frontal and parietal lobes (green triangle), the internal bilateral thalamus, bilateral caudate nucleus head (white arrows), and the periaqueductal area at the posterior margin of the brainstem. (G, H, and I) did not show clearly changes in the same regions. Scale bar = 1 cm. DWI = diffusion-weighted imaging, MRI = magnetic resonance imaging.

On the 9th day, cerebral MRI showed bilateral thalamic, paraventricular, brainstem periaqueductal, and left parietal lobe hyperintensity on T2 FLAIR (Fig. 1B and C) and DWI (Fig. 1D–F). T1-weighted (Fig. 1A) and enhanced MRI (Fig. 1J–L) did not demonstrate any abnormalities. On the tenth day, ASL indicated increased CBF in bilateral frontal/parietal paramedian regions (Fig. 2A), bilateral thalamus, and brainstem periaqueductal areas (Fig. 2B and C). Repeat DWI/ADC revealed multifocal hyperintense lesions in bilateral frontal/parietal lobes, thalamus, paraventricular regions, midline-adjacent areas, medulla, and brainstem periaqueductal zones (Fig. 2D–F), with lower ADC (e.g., 839 ± 35.27* × 10⁻⁶ mm²/s* in thalamus, Fig. 2G–I). Head/neck MRA was unremarkable. These findings are consistent with cytotoxic edema, reflecting intracellular water accumulation due to ATP depletion.

The patient was administered vasoactive agents, underwent fluid resuscitation, received corticosteroids, gastroprotection, bronchodilators, leukocyte boosters, and nutritional support. On the 11th day, she deteriorated to a comatose state. Despite the medical team’s recommendation for further hospitalization pending thiamin testing, the family chose to transfer her to a local hospital. Post discharge laboratory analysis confirmed thiamin deficiency, with her serum vitamin B1 level measuring < 1 ng/mL (reference range: 1–10 ng/mL). This, combined with a history of vomiting and MRI findings, led to the diagnosis of WE. Urgent intravenous thiamin replacement was strongly advised by the medical team during a follow-up telephone consultation.

## 3. Discussion

This case demonstrates the swift advancement of WE, characterized by rapid development of extensive bilateral lesions within a 24-hour period, as evidenced by DWI and ADC scans. ASL results revealed hyperperfusion in regions that subsequently exhibited diffusion restriction, indicating potential early perfusion abnormalities preceding cytotoxic edema.

The clinical presentation of WE often lacks specificity and may be incomplete, leading to frequent delays in diagnosis. While the classic triad of encephalopathy, oculomotor dysfunction, and ataxia is traditionally linked with WE, it is present in fewer than 10% of cases.^[13]^ In the case discussed, initial symptoms manifested as apathy and decreased responsiveness, progressing rapidly to stupor and coma within 4 days. Due to the patient’s unresponsiveness, neurological examination was limited, with no evident oculomotor or ataxic abnormalities observed. Such atypical manifestations are well-documented and underscore the diagnostic challenge, especially in nonalcoholic and oncology patients.

Laboratory findings yielded limited diagnostic insight. In contrast to Bhan et al’s observations of unremarkable serum biochemistry and CSF results in a WE patient,^[10]^ our case presented mild cytopenia, possibly due to chemotherapy, and a slight increase in CSF protein, alongside generally unremarkable CSF metrics. The lack of notable deviations in serum electrolytes, renal and hepatic profiles, and CSF cell counts further emphasizes the constrained efficacy of standard laboratory assessments in WE diagnosis.

Bhan et al also described low-density changes in the bilateral thalami on CT and corresponding hyperperfusion on CT perfusion (CTP), despite normal findings on CT angiography.^[10]^ MRI remains the most sensitive modality for diagnosing WE, with typical findings including symmetrical T2/FLAIR hyperintensities in the thalami, mammillary bodies, periaqueductal gray, and 4th ventricular floor. Signal abnormalities may also involve the cerebellum, midbrain, basal ganglia, and corpus callosum.^[4,5]^ DWI commonly reveals restricted diffusion in the mammillary bodies,^[14]^ thalamus, periventricular region,^[15]^ and midbrain,^[16]^ although ADC signals vary across reports – ranging from high to low or even normal intensity.^[6,7,15–17]^

In our case, by the 9th day of hospitalization, MRI findings displayed hyperintensities on T2-FLAIR and DWI in the bilateral thalami, periventricular areas, and periaqueductal gray matter, accompanied by corresponding ADC hypointensity indicative of cytotoxic edema. Subsequent DWI within 24 hours revealed swift lesion enlargement, encompassing the bilateral frontal and parietal lobes, medulla oblongata, and midline areas, highlighting the rapid advancement of WE.

ASL perfusion imaging revealed a significant increase in CBF within and beyond the regions corresponding to DWI lesions, particularly in the frontal and parietal lobes adjacent to the midline. The discrepancy between perfusion and diffusion patterns suggests that alterations in microcirculation may precede the development of cellular edema. While previous ASL studies on WE, such as the work by Ann et al,^[11]^ have documented hyperperfusion in the medial thalami and periaqueductal gray matter, our case represents the first instance of extensive ASL hyperperfusion in cortical areas surpassing the DWI lesion volume. Normal findings on enhanced MRI and MRA indicate the absence of significant large-vessel pathology or disruption of the blood-brain barrier, supporting the proposition that the ASL findings reflect localized microvascular dysfunction rather than structural vascular abnormalities.

**Figure 3.**
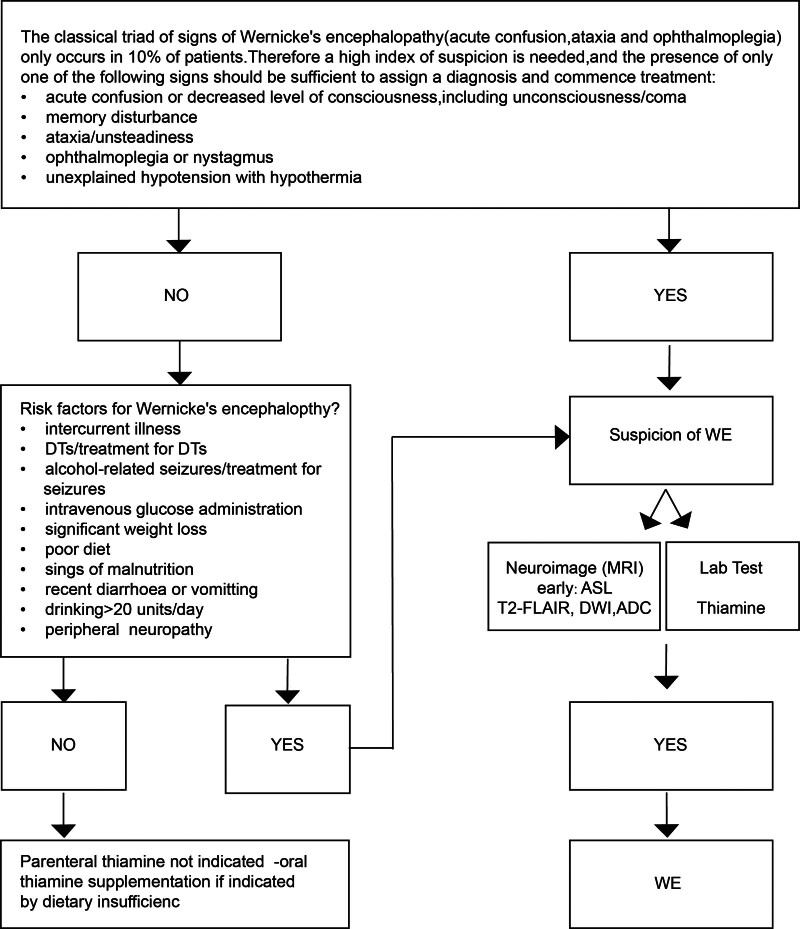
Diagnostic flowchart illustrating the decision-making process in Wernicke encephalopathy. This schematic highlights the clinical decision-making pathway from symptom suspicion to neuroimaging and thiamin therapy, reinforcing the principle of early empirical treatment in high-risk patients. WE = Wernicke encephalopathy.

Figure 3 presents a diagnostic and treatment flowchart for WE, reflecting current consensus and expert recommendations. The diagram emphasizes the need for high clinical suspicion, even in the absence of the classical triad of encephalopathy, ophthalmoplegia, and ataxia – which occurs in fewer than 10% of cases. It guides clinicians through a structured algorithm based on symptom presentation, risk factor assessment, neuroimaging (including ASL, DWI, and T2-FLAIR), and laboratory testing (thiamin levels). Importantly, the chart supports empirical thiamin administration in high-risk patients without waiting for confirmatory results. This case aligns with the flowchart structure, as early ASL abnormalities and evolving DWI lesions illustrated the dynamic pathophysiology of WE, despite initially atypical clinical features.

Research conducted by Watanabe et al in animal models has revealed comparable microcirculatory abnormalities associated with thiamin deficiency, such as astrocytic and oligodendrocytic swelling, petechial hemorrhage, and perivascular necrosis.^[12]^ These observations align with the proposed mechanism in our study, suggesting that the heightened perfusion detected through ASL imaging could indicate a condition of “luxury perfusion” stemming from autoregulatory dysfunction and metabolic strain.

A significant limitation of this study is the lack of intravenous thiamin administration and subsequent imaging follow-up. The absence of post treatment MRI or ASL impedes our ability to evaluate lesion reversibility or establish a causal relationship between perfusion irregularities and cytotoxic damage. Furthermore, this study is based on a single-patient case, underscoring the necessity for validation in larger prospective investigations incorporating sequential imaging assessments and clinical correlations.

In contrast to previous studies, exemplified by Ann et al, which identified ASL hyperperfusion in the medial thalami and periaqueductal gray, this case exhibited pronounced hyperperfusion in the bilateral frontal and parietal lobes. Notably, there is no existing literature documenting ASL-detected perfusion abnormalities in these cortical regions surpassing the degree of restricted diffusion.

These results indicate that ASL could function as a precise indicator for initial cerebral microcirculatory dysfunction in WE, offering the potential for timelier detection compared to traditional diffusion imaging. This broadens the diagnostic capacity of ASL and underscores the significance of integrating perfusion imaging into standard MRI protocols in cases of suspected WE, particularly in individuals with vague clinical symptoms.

## Acknowledgments

This work was supported by the China Postdoctoral Science Foundation 2021M690790.

## Author contributions

**Conceptualization:** Zhigang Li, Lihui Zhu.

**Formal analysis:** Jiadi Chen.

**Investigation:** Qicheng Min, Jiadi Chen, Juxiang Su.

**Visualization:** Boyong Chen, Wenming Chen.

**Writing – original draft:** Zhigang Li.

**Writing – review & editing:** Lihui Zhu.
